# Analysis of the chloroplast genome and phylogenetic evolution of *Bidens pilosa*

**DOI:** 10.1186/s12864-023-09195-7

**Published:** 2023-03-14

**Authors:** Danchun Zhang, Jiajun Tu, Xiaoxia Ding, Wan Guan, Lu Gong, Xiaohui Qiu, Zhihai Huang, He Su

**Affiliations:** 1 Guangdong Provincial Hospital of Chinese Medicine, Guangzhou, Guangdong 510006 China; 2 The Second Clinical College of Guangzhou University of Chinese Medicine, Guangzhou, Guangdong 510006 China; 3 Luqiao Hospital, Taizhou Enze Medical Center (Group), Taizhou, 318050 Zhejiang China; 4 Key Laboratory of Quality Evaluation of Chinese Medicine of the Guangdong Provincial Medical Products Administration, Guangzhou, Guangdong 510000 China

**Keywords:** *Bidens pilosa*, *Bidens bipinnata*, *Bidens alba*, Chloroplast genome, Phylogeny

## Abstract

Chloroplast genomes for 3 *Bidens* plants endemic to China (*Bidens bipinnata* Linn., *Bidens pilosa* Linn., and *Bidens alba* var. *radiata*) have been sequenced, assembled and annotated in this study to distinguish their molecular characterization and phylogenetic relationships. The chloroplast genomes are in typical quadripartite structure with two inverted repeat regions separating a large single copy region and a small single copy region, and ranged from 151,599 to 154,478 bp in length. Similar number of SSRs and long repeats were found in *Bidens*, wherein mononucleotide repeats (A/T), forward and palindromic repeats were the most in abundance. Gene loss of *clpP* and *psbD*, IR expansion and contraction were detected in these *Bidens* plants. It seems that *ndhE*, *ndhF*, *ndhG*, and *rpl32* from the *Bidens* plants were under positive selection while the majority of chloroplast genes were under purifying selection. Phylogenetic analysis revealed that 3 *Bidens* plants clustered together and further formed molophyletic clade with other *Bidens* species, indicating *Bidens* plants might be under radiation adaptive selection to the changing environment world-widely. Moreover, mutation hotspot analysis and in silico PCR analysis indicated that inter-genic regions of *ndhD-ccsA, ndhI-ndhG, ndhF-rpl32, trnL_UAG-rpl32, ndhE-psaC, mat*K*-rps16, rps2-atpI, cemA-petA, petN-psbM* were candidate markers of molecular identification for *Bidens* plants. This study may provide useful information for genetic diversity analysis and molecular identification for *Bidens* species.

**Supplementary Information:**

The online version contains supplementary material available at 10.1186/s12864-023-09195-7.

## Introduction

The genus *Bidens* from Asteraceae family comprises approximately 230 species, which distributed from the tropics to subtropics in Asia, America and other continents [1]. *Bidens* plants have attracted researchers’ attention from ecologist, pharmacologist and biologist. For example, Sun et al. [2] found *Bidens pilosa* could tolerate high-cadmium (> 8 mg/kg) soil environment and accumulated to more than 100 mg/kg concentration of cadmium (Cd) in stems, leaves and shoots at the flowering and mature stages, furthermore, *Bidens* species suggesting it is a Cd-hyperaccumulator which is promising plant in Cd-polluted soil recovery. Phytochemical investigation of *Bidens pilosa* indicating compounds like polyacetylenes, chalcone glucoside, flavonoid, triterpenoids et al. might be bioactive components which contribute to therapeutic effects in anti-inflammatory, analgesic, antibacterial reported in *Bidens* plants [3–8]. Because of displaying highly ecological and morphological diversity, occupying a wide of habitats including sand dunes, deserts, lava flows, mesic forests, rainforests, scrublands, and wetland bogs, *Bidens* species especially for *Hawaiian Bidens* radiation has attracted biologist’ attention for a long time and this genus has been one of the best examples of adaptive radiation in the *Hawaiia* flora [9]. Based on the study reported by Knope et al. [10], *Hawaiian Bidens* radiation occurred just within the last 2 million years. Relative short-time divergency and great niche difference among *Bidens* species caused low levels of genetic variation and high levels of morphological variation, resulted hindered usage of classic DNA markers and morphological markers in resolving the identification and their evolutionary histories. To the best of our knowledge, the classification of *Bidens* is still controversial, more constructive studies remain to be started to fulfill the gap between current knowledge about evolutionary history and the real situation.

The reason for classical DNA markers such as *mat*K, *psb*A*-trn*H, ITS1 and ITS2 are hard to separate closely-related species is that they are usually several hundred of base pairs in length, therefore limiting genetic variation information which is necessary for reaching certain resolution. Based on the records on Flora of China, there are 10 species *Bidens* species distributed in China [11], what is interesting is that similar morphological and chemical characteristics were found which hindered the studies about both discrimination and phylogenetic history for those species [12, 13]. The first report about molecular identification for *Bidens* species was conducted by Tsai et al. [12], who applied the noncoding regions from chloroplast genome (*trn*L intron and *trn*L*-trn*F) and nuclear ribosomal DNA (ITS1, 5.8S and ITS2) to *Bidens* species identification, the results suggested ITS1, 5.8S, ITS2 and *trn*L intron could only separate *B. biternata* from *B. pilosa* var. *pilosa*. In our preliminary experiments, we retrieved all available ITS1 and ITS2 sequences from NCBI nucleotide database and found that neither ITS1 nor ITS2 could generate reasonable resolution for *Bidens* species separation (Figs. S1 and S2).

The chloroplast (CP) is an important organelle relating to photosynthesis, and has an independent circular genome with typical quadripartite structure. In angiosperms, CP genomes are highly conserved in gene composition and usually uniparental inheritance, and have low nucleotide substitution rate [14]. Generally, CP genomes are > 100 kb in length, which make CP genomes have significant higher capacity for storing genetic variation than that in widely-used DNA barcodes such as *mat*K, *trn*H*-psb*A, ITS1 and ITS2, with only several hundred base pairs in length. In addition, CP genomes are high in copy number in plant cells, for example, about 1000 copies in *Arabidopsis thaliana* leaf cell was reported [15]. In the past decade, a large number of studies have reported that CP genome is valid tool both in closely-related species identification and in phylogenic-history tracing. For example, Gong et al. [16] unraveled the phylogenetic relationships and hybridization history for *Amomum* species and developed novel DNA markers for species identification. Song et al. [17] re-constructed *Styrax* species phylogeny and developed specific DNA barcodes which exhibit higher discriminatory power. Cao et al. [18] reported unique variable sites in CP genes, *ndh*F, *rpl22* and *ycf1*, which could be applied to distinguish *Viola philippica* from all other *Viola* samples including the most closely related species. Based on the previous reports about CP genome and its successful application in species identification, we hypothesized that it might be an effective method to generate reasonable resolution for *Bidens pilosa* Linn., *Bidens bipinnata* Linn. and *Bidens alba* var. *radiata* and to provide more sound information about phylogeny history of *Bidens* species.

In this study, we reported the CP genomes of *Bidens pilosa* Linn., *Bidens bipinnata* Linn. and *Bidens alba* var. *radiata*. Then we conducted CP genome-wide comparative analysis among those species with other published available species in *Bidens*. Our main goals were: (1) to explore the molecular characteristics of those three chloroplast genomes; (2) making comparison CP genome-widely to detect structural variation among *Bidens* plants. (3) to analyze sequence variations, get highly divergent regions and develop specific DNA markers for *Bidens* species identification. (4) unraveling the evolutionary history and explore the phylogenetic relationships of *Bidens*. This study will provide but not limited to useful information to in clarifying species identification and phylogenetic history about *Bidens* plants.

## Materials and methods

### Plant materials, DNA extraction and sequencing

Fresh and healthy samples for *Bidens pilosa* Linn., *Bidens bipinnata* Linn. and *Bidens alba* var. *radiata* were collected at Xiaoguwei County, Guangzhou city, Guangzhou province, China. The leaves were stored in the liquid nitrogen immediately after being removed from the plants and transferred to -80 ℃ refrigerator (Eppendorf, Hamburg, Germany) right back to the laboratory. All samples were identified by Ye YuShi, the engineer in South China Botanical Garden, Chinese Academy of Sciences, and all voucher specimens were deposited in the Second Clinical College of Guangzhou University of Chinese Medicine (voucher ID numbers: BD190529S1 for *Bidens pilosa*, BD190529S2 for *Bidens bipinnata* and BD190529S3 for *Bidens alba*).

Genomic DNA was extracted using a DNA easy Plant Mini Kit (Qiagen Co., Hilden, Germany) following the manufacturers' instructions. The quality and quantity of DNA was determined with 1% gel electrophoresis and Nanodrop2000C (ThermoScientific, Delaware, USA). The DNA was fragmented into 400 ~ 600 bp by Covaris sonication (Covaris M220, Woburn, MA, USA) and thereafter applied to sequencing library construction with the NEBNext® Ultra™ DNA Library Prep Kit Illumina (New England, Biolabs, Ipswich, MA, USA). Libraries were sequenced by Illumina HiSeq4000 platform (Illumina Inc. CA, USA).

### Chloroplast genome assembly and annotation

We handled our sequencing reads according to the method published by Zhou et al. [19], the quality of raw reads was evaluated by FastQC (v0.11.9) software [20], low-quality reads were filtered and trimmed by Trimmomatic (v0.39) [21]. Chloroplast-like (CP-like) reads were extracted by blast the clean reads against the collection of CP genomes retrieved from NCBI nucleotide database. After which, CP-like reads were assembled to continuous contigs by SOAPdenovo2 [22] and scaffolded by SSPACE [23], finally, the remaining gaps were filled by Gap Filler package [24].

We predicted and annotated the CP-genomes in two-round steps. Firstly, the assemblies were predicted and annotated by CPGAVAS2 [25] with default parameters, except for taking 2544-genome-model rather than 43-genome-model as reference dataset. Secondly, gene model generated from above were manually curated by Apollo software [26] with supporting information generated by blast searches against Swiss-Prot database. Finally, the original CPGAVAS2’s prediction was updated by the latest GFF3 file. The chloroplast genome were visualized with OGDRAW v1.3.1 [27].

In addition, tRNAs were predicted by tRNAScan-SE software [28]. Relative synonymous codon usage (RSCU) for protein coding genes was calculated by CodonW (http://codonw.sourceforge.net) [29]. Simple sequence repeats (SSRs) analysis was conducted with MISA v2.1 [30], as to long repeats, an online tool REPuter was used for identifying forward (F), palindromic (P), reverse (R), and complement (C) repeats with default parameters[31]. GC content was calculated with GC function planted in sequin package [32].

### Analysis for boundary regions of CP-genomes

CP genomes of *B. pilosa*, *B. bipinnata* and *B. alba* and other three species, *B. asymmetric*, *B. campylotheca*, and *B. cervicate*, retrieved from NCBI were thoroughly compared and analyzed at genome-level. In details, contraction and expansion of IR regions among LSC, IRb, SSC and IRa were visualized by IRScope [33]. MAFFT [34] was applied to multiple sequence alignment (MSA) for CP genomes. Nucleotide diversity (Pi) was calculated with DnaSP [35] taking the MSA file as input, the step size and sliding window length were set to 200 bp and 800 bp respectively. In addition, in order to find available molecular markers for species identification, mVISTA [36] was used for CP comparative analysis and visualization, by which highly divergent regions were detected. Candidate molecular markers were verified by in silico PCR with Fast-PCR.

### Phylogenetic analyses and selective pressure evaluation

A phylogenetic tree was reconstructed with the ML-based method with RAxML-ng [37] by providing multiple sequence alignment files generated from whole chloroplast genomes with MAFFT. In detail, there were 15 species were used in the phylogenetic analysis, and *H. annuus* was regarded as an outgroup. For RAxML-ng, parameters for the model were set to “GTR + R4 + FO”, bs-metric was set to “fbp, tbe”, and 100 starting trees (50 random and 50 parsimony-based) were used to pick the best-scoring topology by setting–tree to “pars, rand” [38]. To evaluate the selection pressure on cp protein-coding genes, we extracted the shared non-redundant genes among species, in which each gene’s CDS-pair of one-by-one species’ combination were extracted and aligned by MAFFT. The rates of synonymous substitutions (Ks) and non-synonymous substitutions (Ka) and Ka/Ks were then calculated by ParaAT2.0 [39], in which KaKs_Calculator [40] is inplanted. The command we applied in this study is “ParaAT.pl –c 11 –h homologs.txt –n CDS –a PEP –p proc –o OUT –k –f axt –m mafft –v”.

## Results

### Genome characteristics

**Fig. 1:**
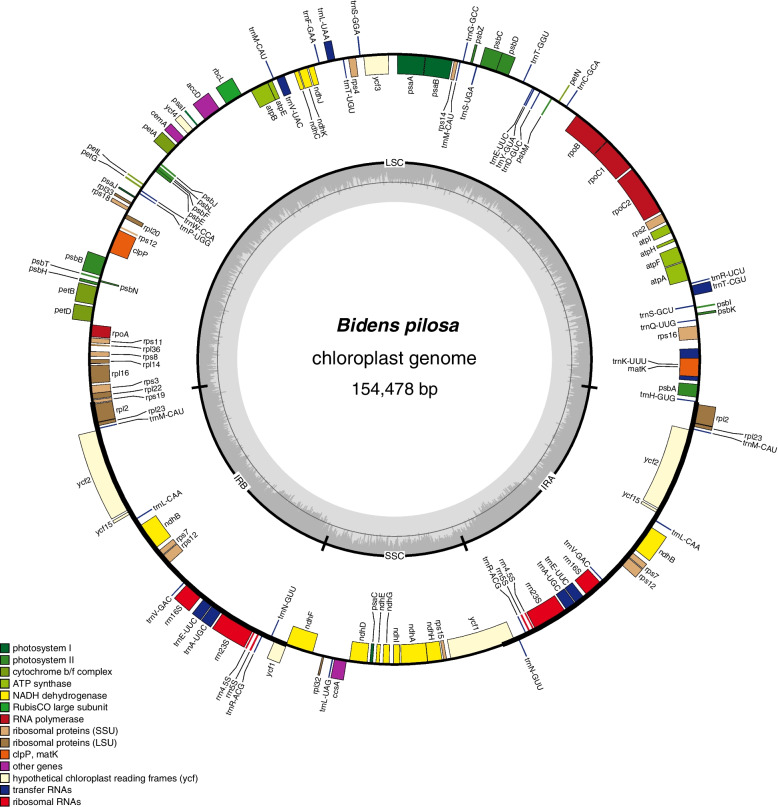
Chloroplast genome map of *Bidens pilosa*

CP genomes for these *Bidens* species are in typical quadripartite structures (Fig. 1, Figs. S3 and S4), consisting of LSC, two IRs and a SSC region, where LSC ranged from 151,599 to 154,478 bp, IRs from 24,245 to 26,264 bp, LSC from 83,856 to 84,240 bp and SSC from 17,780 to 18,439 bp, respectively. The sizes of CP genome for those species varied little and are 154,478, 151,611 and 151,599 bp for *B. pilosa*, *B. bipinnata* and *B. alba* (Table 1). GC content are unevenly distributed, i.e., GC contents for LSC, SSC, and IR and CP genome for *B. pilosa* are 35.64%, 31.34%, 42.42% and 37.52%, *B. bipinnata* are 35.59%, 30.97%, 43.21% and 37.48%, and *B. alba* are 35.56%, 30.97%, 43.14% and 37.48%. A total of 129, 129 and 130 genes are found in *B. pilosa*, *B. bipinnata* and *B. alba*, respectively, wherein 86, 85 and 86 are protein-coding genes, 35, 36 and 36 are tRNA genes, in addition the remaining 8 genes for those species are rRNA genes. There are 16, 14, and 15 intron-containing genes in *B. pilosa*, *B. bipinnata*, and *B. alba*, respectively, in which there are 6 intron-containing tRNA genes (trnA-UGC, trnC-ACA, trnE-UUC, trnK-UUU, trnL-UAA, and trnS-CGA) and 12 intron-containing genes (Table S1).

**Table 1:** Characteristics of the CP genomes

Species	*Bidens pilosa*	*Bidens bipinnata*	*Bidens alba*
Total length(bp)	154,478	151,611	151,599
LSC.length(bp)	84,170	84,240	83,856
SSC.length(bp)	17,780	18,439	18,439
IR.length(bp)	26,264	24,245	24,652
CDS.length(bp)	76,044	76,674	77,109
CDS length/Total length (%)	49.23	50.57	50.86
Genes number	129	129	130
Protein-coding genes	86	85	86
tRNA genes	35	36	36
rRNA genes	8	8	8
Intron-containing genes	16	14	15
LSC.GC (%)	35.64	35.59	35.56
SSC.GC (%)	31.34	30.97	30.97
IR.GC (%)	42.42	43.21	43.14
Total GC (%)	37.52	37.48	37.46
CDS.AT1 (%)	54.49	54.45	54.41
CDS.AT2 (%)	61.90	61.85	61.83
CDS.AT3 (%)	70.15	70.11	70.09

As to the protein-coding genes, 5 genes responding for photosystem I (*psaA, B, C, I, J*)*,* 15 genes for photosystem II (*psbA*,* B*,* C*,* D*,* E*,* F*,* H*,* I*,* J*,* K*, *M*,* N*,* T*,* Z*, *ycf3*)*,* 6 genes for ATP synthase (*atpA*,* B*,* E*,* F*,* H*,* I*)*,* 11 genes for large ribosomal proteins (*rpl2, 14, 16, 20, 22, 23, 32, 33, 36*)*,* and 12 genes for small ribosomal proteins (*rps2, 3, 4, 7, 8, 11, 12, 14, 15, 16, 18, 19*) were found for *B. pilosa*, *B. bipinnata* and *B. alba*, Wherein *rpl2, 23, rps12* are duplicated genes, *clpP* is absent from *B. bipinnata* but kept in *B. pilosa* and *B, alba* and *psbD* only kept in *B. pilosa* but absent from *B. alba* and *B. bipinnata* (Table 2).

**Table 2:** Common genes annotated in the cp genomes

Category of genes	Group of genes	Name of genes	number
photosynthesis	Photosystem I	*psaA, psaB, psaC, psaI, psaJ*	5
	Photosystem II	psbA, psbB, psbC, psbD^*,&^, psbE, psbF, psbH, psbI, psbJ, psbK, psbM, psbN, psbT, psbZ, ycf3^*^	15
	NADH-dehydrogenase	*ndhA, ndhB(*× 2)**, ndhC, ndhD, ndhE, ndhF, ndhG, ndhH, ndhI, ndhJ, ndhK	12
	Cytochrome b/6f complex	petA, petB^*^, petD, petG, petL, petN	6
	ATP synthase	atpA, atpB, atpE, atpF^*^, atpH, atpI	6
	Rubisco	*rbcL*	1
Self replication	Large subunit of ribosome	rpl2(× 2)**, rpl14, rpl16, rpl20, rpl22, rpl23(× *2), rpl32, rpl33, rpl36*	11
	Small subunit of ribosome	* rps2, rps3, rps4, rps7(× 2), rps8, rps11, rps12(*× 2), rps14, rps15, rps16*, rps18, rps19	14
	DNA dependent RNA polymerase	rpoA, rpoB, rpoC1^*^, rpoC2	4
	rRNA genes	*rrn16S(*× *2), rrn23S(*× *2),rrn4.5S(*× *2),rrn5S(*× *2)*	8
	tRNA genes	*trnA-UGC(*× 2)**, trnC-GCA^*^, trnD-GUC, trnE-UUC(× 3)^*^, trnF-GAA, trnG-GCC, trnH-GUG, trnK-UUU^*^, trnL-CAA(× 2), trnL-UAA^*^, trnL-UAG, trnM-CAU(× *4),trnN-GUU(*× *2),trnP-UGG, trnQ-UUG, trnR-ACG(*× 2),trnR-UCU, trnS-CGA^*^,trnS-GCU,trnS-GGA, trnS-UGA, trnT-UGU, trnV-GAC(× *2),trnW-CCA,trnY-GUA,(trnT-GGU*^*#*^*)*	35
Other genes	accD, ccsA, cemA, (clpP^*, @^*), infA, mat*K		5
Unknown function	*ycf1, ycf2(*× 2)**, ycf4, ycf15(× *2)*		6

^*^ Indicates genes containing introns, ** indicates genes containing two introns

^#^absent from *B. pilosa*, ^@^absent from *B. bipinnata*, ^&^only exist in *B. pilosa*

(× 2) indicates that the gene sequence was repeated twice

### Relative synonymous codon usage

Relative Synonymous Codon Usage (RSCU) for the protein-coding genes of CP genomes for *B. pilosa*, *B. bipinnata* and *B. alba* was calculated by CodonW on the basis of protein-coding genes (Table S2). There are 64 types of codons encoding 20 amino acids were found in this study, in which 6 codons encode Serine (Ser), Leucine (Leu), and Arginine (Arg), 4 codons encode Alanine (Ala), Glycine (Gly), Proline (Pro), Threonine (Thr), 3 codons encode Isoleucine (Ile), 1 codon encode Methionine (Met) and Tryptophan (Trp), respectively, the remaining amino acids were encoded by 2 codons. In addition, we found Leucine was the amino acid in the highest frequency ranging from 10.6% to 10.7% while Cysteine (Cys) was in the lowest ranging from 1.11% to 1.13% in CP genome protein-coding genes. Generally, codon with RSCU > 1 when encoding an amino acid should be regarded as codon usage bias preference [29, 41]. As there is only one codon encoding Methionine and Tryptophan respectively, RSCU for codon of these amino acid was 1. About half of the codons have RSCU > 1 (30/64), 29/30 of which end with base A or T. Moreover, Leu, Arg and Ser are amino acids with the highest RSCU value both for the 3 *Bidens* plants, exhibiting usage bias for tta, aga, tct, gct with RSCU value of 1.88, 1.87, 1.78 for *B. pilosa*, 1.88, 1.84, 1.82 for *B. bipinnata*, 1.87, 1.85, 1.81 for *B. alba* respectively (Table S2).

### IR contraction and expansion

**Fig. 2:**
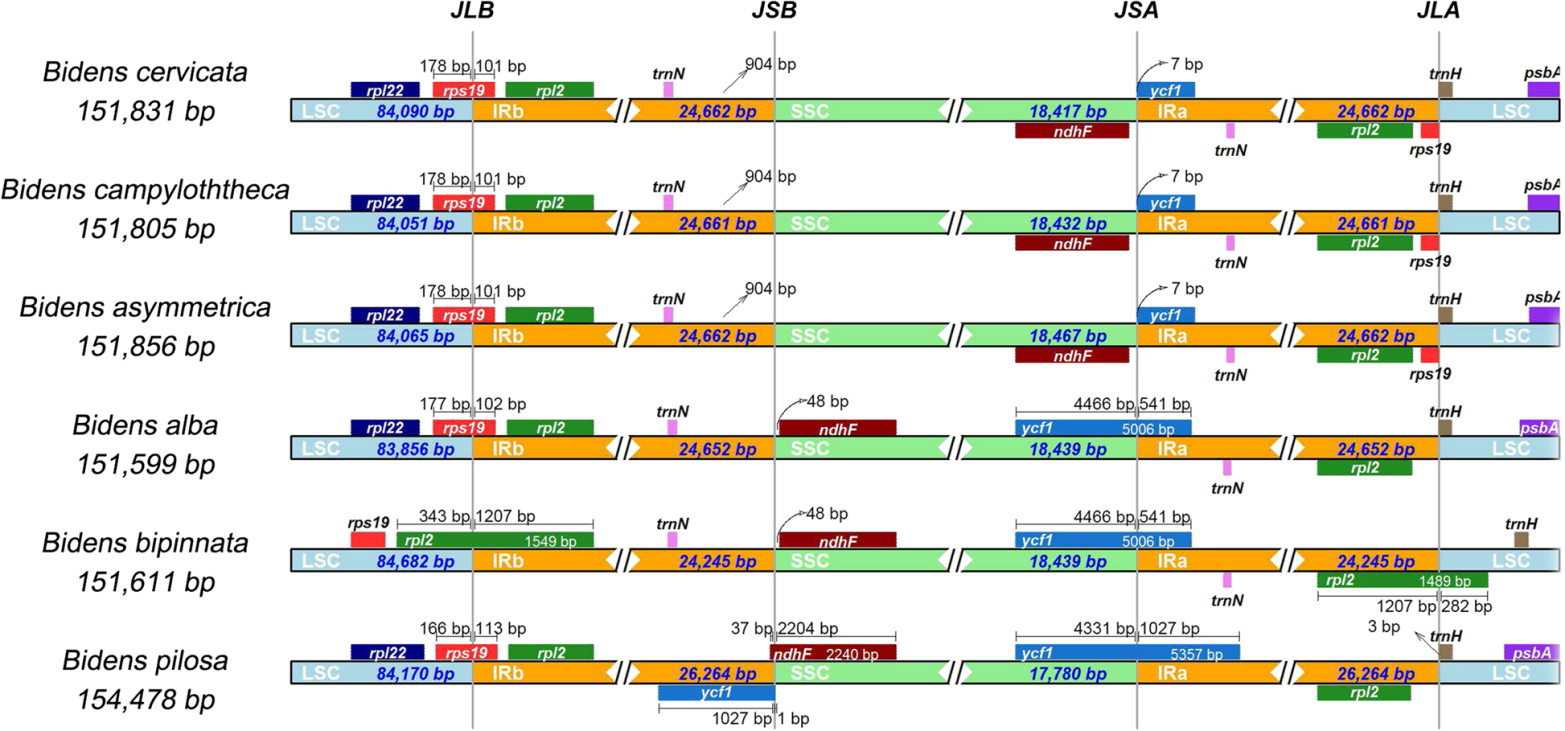
Comparison of the boundaries of the LSC, SSC and IR regions. JLB: junction between LSC and IRb; JSB: junction between SSC and IRb; JSA: junction between. SSC and IRa; JLA: junction between LSC and IRa

The length of IR from *Bidens* species we have sequenced varied in length, is 24,652, 24,245, 26,264 bp for *B. alba, B. bipinnata* and *B. pilosa*, respectively. It is more divergent than that of published species (ranging 24,661 to 24,662 bp), indicating IR expansion might occurred in *B. pilosa* while contraction might occurred in *B. bipinnata*. In addition, contraction and expansion of IR boundary contributes to genome size variations for CP genomes [13, 16, 42]. 3 CP genomes assembled in this study and 3 other *Bidens*’ published CP genomes (*B. cervicata*, *B. asymmetrica* and *B. campylotheca*) were included in comparison of IRs regions at four junctions between two IRs (IRa and IRb) and the two single-copy region (LSC and SSC) (Fig. 2). As a result, *rps19* genes located at the junctions of IRa/LSC and LSC/IRb, wherein, 166 to 178 bp located at LSC region and 101 to 113 bp located at IRb region. As for *rpl2*, the whole gene located at IR regions in all *Bidens* species except for *B. bipinnata* where 282 to 343 bp located at LSC region. As for *ndh*F in *Bidens* plants, located at SSC region, variation of location in SSC was found that *ndh*F located close to IRb region in the three newly assembled CP genomes (specifically, it crossed IRb and SSC in *B. pilosa*) while close to IRa region in other 3 species. In addition, it is noteworthy to mention *ycf1*, which spanned SSC/IRa junction in *B. pilosa*, *B. bipinnata* and *B. alba* while only located at IRa in *B. cervicata*, *B. campylotheca*, and *B. asymmetrica*. Those results suggesting genome structure varied in *Bidens* species.

### Comparative chloroplast genomic analysis

**Fig. 3:**
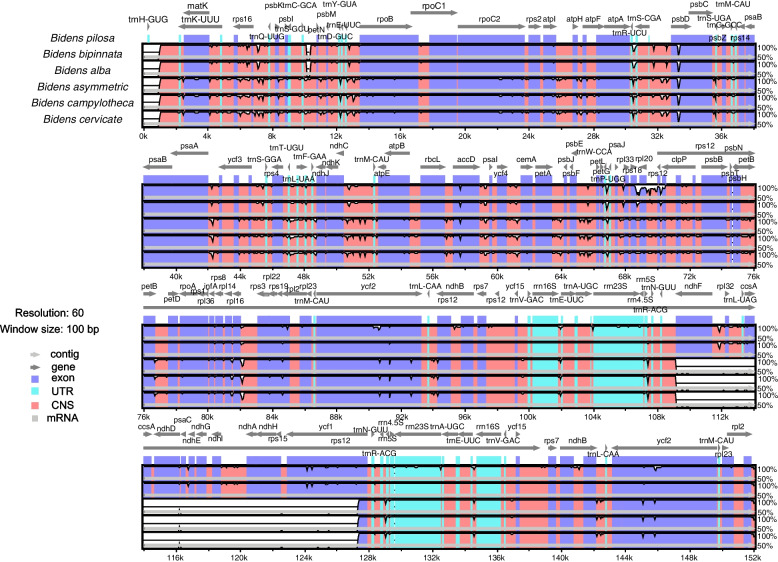
Comparison of five chloroplast genomes using mVISTA by taking *B. pilosa* sequence as a reference. The lower left corner is the color coding of gene function, grey arrows indicate the orientation of genes, red bars represent conserved non-coding sequences, purple bars represent exons, and blue bars represent introns. The y-axis represents the percentage identity (shown: 50–100%)

Genome divergence and sequence identity were calculated using the mVISTA by taking *B. pilosa* as reference (Fig. 3). The results indicate that the LSC and SSC regions are more divergent than IR regions, this finding is in accordance to previous studies [13, 16, 42]. The results revealed that most of the variation was located in the LSC and SSC regions, and slight variation occurred in the IR regions, whereas the coding regions were more conserved than the non-coding regions. The CP genomes of the three *Bidens* species in this study more conservative than those of the other three species due to the detection of blank regions. We also found slight differences for *rps12* and *ycf2*, which exist in multiple fragments among six species. Moreover, the highly divergent non-coding regions among the six CP genomes appear in the intergenic spacer regions (IGS), including *ndhE-psaC*, *ndhE-ndhG*, *trnL_UAG-rpl32, ndhF-rpl32, ndhI-ndhG*, and *ndhD-ccsA*. Among them, the most divergent coding regions are the *rpl20, ndhA* and *mat*K genes.

### Simple sequence repeat and long repeats analysis

**Fig. 4:**
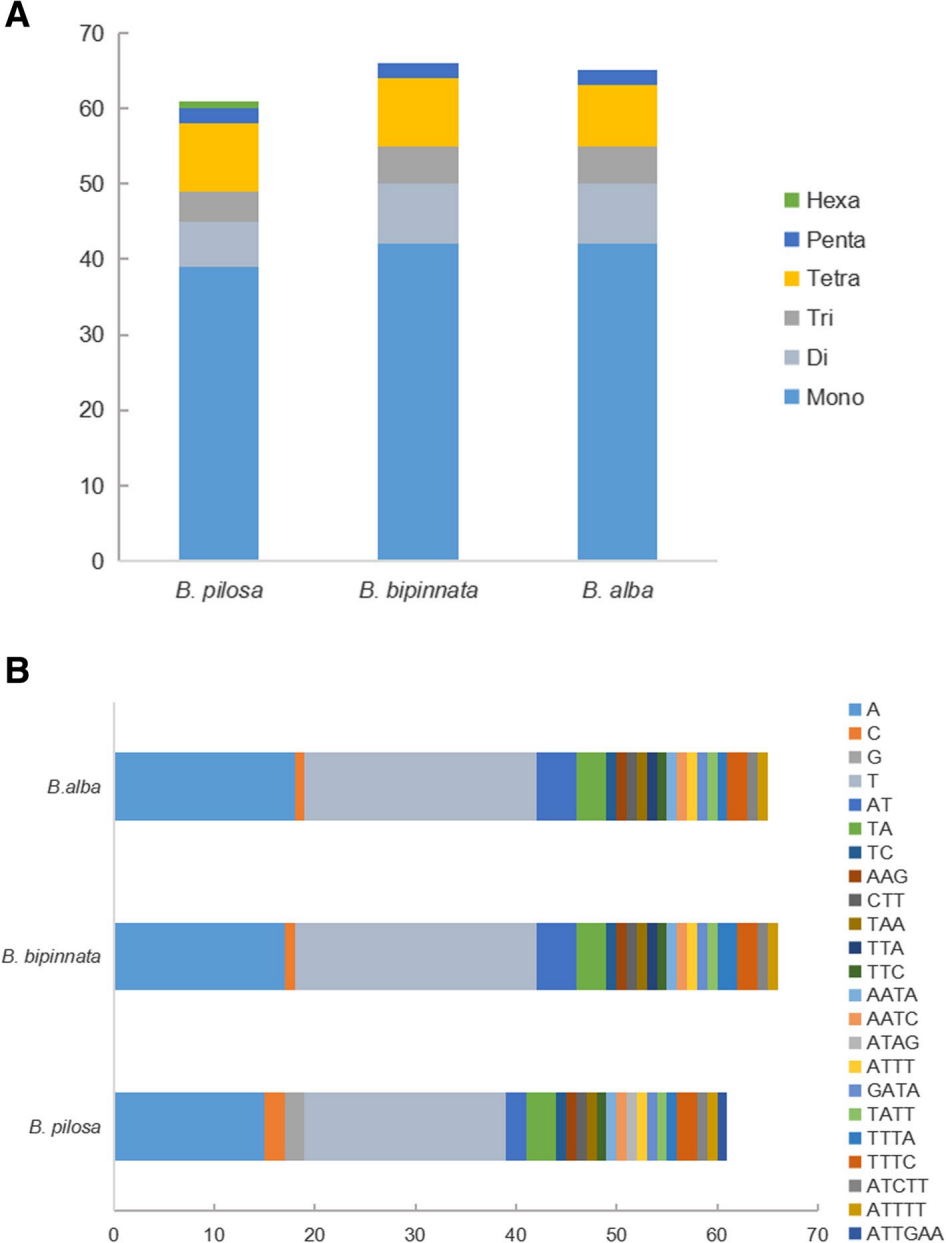
Analysis of simple sequence repeats (SSRs) in the *Bidens* chloroplast genomes. **A**. different SSR types detected in three genomes. **B**. frequency of identified SSR motifs in different repeat class types

**Fig. 5:**
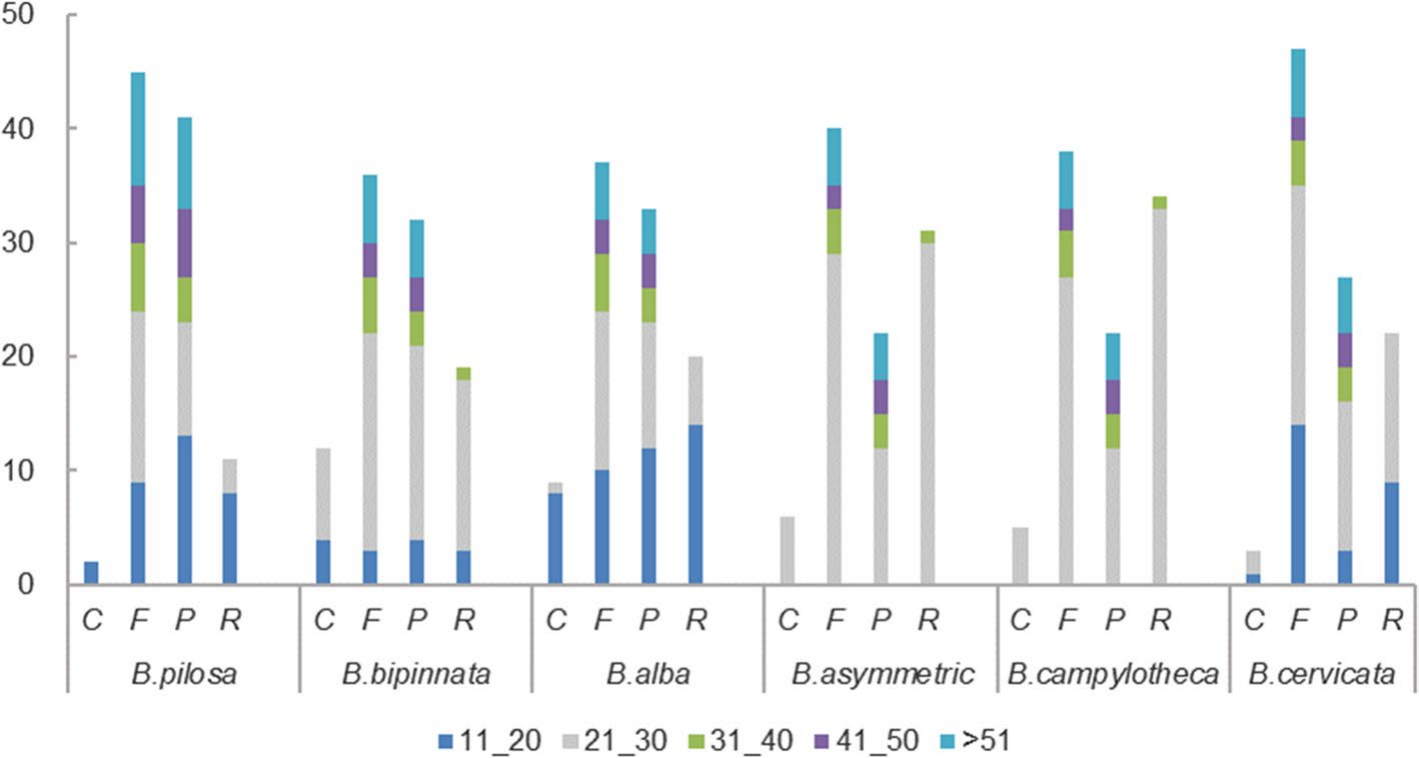
Analysis of repeated sequences in *Bidens* chloroplast genomes. C, F, P and R indicate the repeat types: complement repeat, forward repeat, reverse repeat and palindromic repeat

Simple sequence repeats (SSRs) are tandem repeat DNA sequences 1 ~ 6 bp in length that have been widely applied as molecular markers in species authentication [43–46]. A total of 61, 65, 66 SSRs are detected in *B. pilosa*, *B. alba* and *B. bipinnata*, among which 39, 42 and 42 are mononucleotides, 6, 8, and 8 are dinucleotides, 4, 5, 5 are trinucleotides, 9, 8, 9 are tetranucleotides, 2, 2, 2 are pentanucleotides, interestingly, only 1 hexanucleotide repeat is found in *B. pilosa* (Fig. 4A-B and Table S3A-B). Noteworthy, mononucleotide repeats (A/T) is the highest in number ranging from 35 (*B. pilosa*) to 41 (*B. bipinnata* and *B. alba*) (Fig. 4B). Moreover, long repeats were characterized by REPuter, 99 repeats were detected from those *Bidens* plants separately. Wherein there are 45, 36, 37 forward repeat, 11, 19, 20 reverse repeat, 41, 32, 33 palindromic repeat and 2, 12, 9 complement repeat respectively. As for 3 other published species, 99 long repeats were identified for *B. asymmetric*, *B. campylotheca*, and *B. cervicata* in which there are 40, 38, 47 forward repeat, 31, 34, 22 reverse repeat, 22, 22, 27 palindromic repeat and 6, 5, 3 complement repeat respectively (Fig. 5). The above results suggested that both SSR and long repeats varied among species, by which novel molecular markers could be developed in *Bidens* plants identification.

### Nucleotide diversity

By analyzing the sliding window of *Bidens* CP genomes using DnaSP software, a high level of variability was observed (Fig. S5A). The nucleotide diversity (Pi) averaged 0.14923 for the 67 protein-coding genes and 46 IGS regions among six *Bidens* species, while IR region averaged 0.00758, LSC region averaged 0.05286 and SSC averaged 0.10930 respectively, indicating IR region is more conserved than LSC and SSC region. Taking Pi ≥ 0.05 as threshold [42], 34 divergent regions were found for IGS, such as *ndhE-psaC* (0.38986), *ndhE-ndhG* (0.35152), t*rnL_UAG-rpl32* (0.34343)*, ndhF-rpl32* (0.33850)*, ndhI-ndhG* (0.32686), and *ndhD-ccsA* (0.31234) (high Pi values > 0.3) (Fig. S5B), these findings were in accordance with the results of mVISTA. These divergent regions are candidate markers might be used as DNA barcods for further phylogenetic analyses and species identification [18, 42]. As to chloroplast genes, Pi of *rpl20*, *ndhA* and *mat*K are 0.02101, 0.01011 and 0.00911 (Fig. S5B) respectively, both higher larger than that of *rbc*L (0.00650), which has been applied as one of classic DNA barcodes (Table S4).

### Specific DNA markers for *Bidens*

In our preliminary study, we retrieved 5 classic DNA barcodes (ITS1, ITS2, *psb*A*-trn*H*, mat*K and *rbc*L) of *Bidens* species from NCBI (nucleotide database) for identification analysis, because only sequences for ITS1 and ITS2 were adequate, *psb*A*-trn*H, *mat*K and *rbc*L were remove from further analysis. As a result, we found neither ITS nor ITS2 could separate *Bidens* species at reasonable resolution (Figs. S1 and S2). Based on divergent regions found above, we designed primers (Table S5) for IGS, including *ndhD-ccsA*, *ndhI-ndhG*, *ndhF-rpl32*, *trnL_UAG-rpl32*, *ndhE-ndhG*, *ndhE-psaC*, *matK-rps16*, *ycf1-trnN_GUU*, *rps2-atpI*, *cemA-petA*, and *petN-psbM* are verified by in silico PCR amplification by FactPCR 6.7 [47], in order to develop novel molecular markers for *Bidens* species. As a result, *ndhD-ccsA*, *ndhI-ndhG*, *ndhF-rpl32*, *trnL_UAG-rpl32*, *ndhE-psaC*, *mat*K*-rps16*, *rps2-atpI*, *cemA-petA*, *petN-psbM* were regarded as candidate markers with high confidence (Table S6).

### Selective pressure analysis

**Fig. 6:**
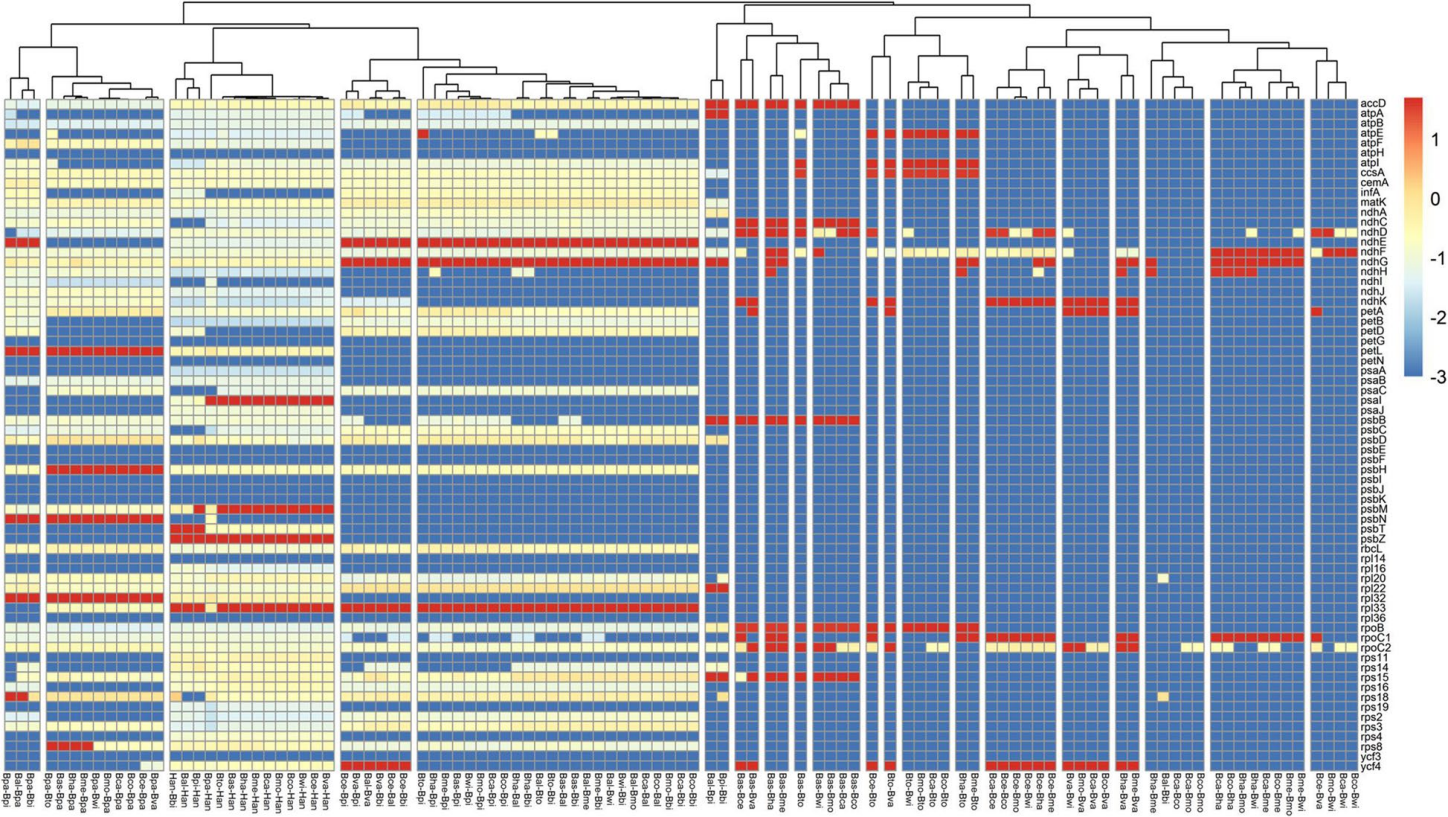
Pairwise Ka/Ks ratios in different genes. The scale factors associated with each value are displayed on the right side of the graph. The color close to red indicates that the gene has a high Ka/Ks ratio. Red indicates positive selection that is significantly enriched, while blue indicates negative selection that is significantly reduced. See Table S7 for abbreviations

We calculated the nonsynonymous (Ka) to synonymous (Ks) substitution ratios for all 68 protein-coding genes of CP genomes from 15 *Bidens* with KaKs_calculator [40] by ‘Ma’ model and statistically tested by Fisher Exact Test(Fig. 6). Overall, Ka/Ks values were less than 0.5 for the majority genes, suggesting that CP genes of the *Bidens* are conserved and mainly under negative selection during the evolution process, which is reasonable for necessary roles played by chloroplast genes and is in accordance with previous studies. In addition, Ka/Ks values of 4 genes (*ndhE*, *ndhF*, *ndhG*, and *rpl32*) were greater than 1, indicating these genes undertaking positive selection pressure during evolution, however, we found only 1 substitution in the multiple sequence alignment (MSA) exist for each gene-pairs, indicating relatively low positive selection occurred to these genes. Further molecular biology studies should be started to evaluate the environmental impact on these genes.

### Phylogenetic reconstruction of *Bidens*

**Fig. 7:**
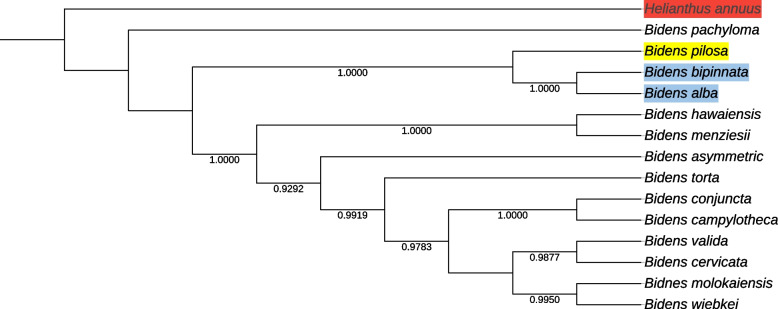
The phylogenetic tree reconstruction base ML

Phylogenetic relationships among 14 *Bidens* species were re-constructed by RaxML-ng software. The phylogenetic tree indicating that the three assemblies of *Bidens* in China are monophyletic and separated from other *Bidens* species (Fig. 7). *B. bipinnata* and *B. alba* were clustered together with supporting rate of 1.0, to which *B. pilosa* was sister group, indicating they are closer related species than others included in this study. Furthermore, all *Bidens* constructed monophyletic relationships indicated they might diverge from the same ancestor and evolved independently around the world. These findings made us confident in proposing a hypothesis that *Bidens* species were indeed highly adaptive to the changing environment world-widely, and were suffering a higher level of radiation adaptive selection compared with that in *Bidens* species occurred at *Hawwii*.

## Discussions

In this study, we collected three *Bidens* species distributed in China and assembled their complete chloroplast genome. We found CP genomes for *Bidens* species varied little in size, but also conserved in genome structure, gene composition and gene order, these findings were in accordance with previous reports that the structure of plastome geome in most angiosperm is generally maternal-inherited and highly conserved [48]. GC content is unevenly distributed at LSC, IR and SSC region, wherein GC content of IR is the highest while the lowest in SSC, higher GC content found in IR region might be resulted by the presence of rRNA (*rrn4.5*, *rrn5*, *rrn16*, and *rrn23*) that has been reported previously in *Asteraceae* cp genomes [49–51]. A CP gene is rarely lost arbitrarily, it is either trasferred to the nuclear genome or functionally replaced by a nuclear gene [52]. Interesting in our study, we found *clpP* was lost from *B. bipinnata* and *psbD* lost from *B. alba* and *B. bipinnata*. Noteworthy, deletion of one intron from *clpP* has been reported in *Vicia sepium *[53]*.*

SSRs have high polymorphism and have been widely used in the study of phylogeny and biogeographic development [54, 55]. Similar number of SSRs and long repeats were found in *Bidens*, while it varied among species in type, which were vastly distributed in the IGS region of LSC, wherein the majority of the SSRs are mononucleotide repeats (A/T) and the majority of long repeats are forward and palindromic repeats, respectively. These information might be useful in developing novel molecular markers. IR expansion and contraction is one of reason causing size variation for CP genome which has been reported in *Polystachya dendrollifora *[42], *Amomum villosum *[16], *Aristolochia debilis *[19], and *Styrax* species [17, 56]. The length of IR varied among *Bidens* species indicated IR expansion and contraction might have been occurred in *B. pilosa* and *B. bipinnata* respectively. Detailed comparison of four IR/SC junctions of *Bidens* species showed that the border structures were highly similar to each another (Fig. 2). Although the boundary regions of the cp genome were relatively stable, we found *rpl22*, *ycf1* located at boundray regions might have been shiffted by IR expansion and contraction.

Because Pi for protein-coding genes and Intergenic regions (IGS) averaged the highest among IR, LSC and SSC, we hypothesized IGS like *ndhD-ccsA, ndhI-ndhG, ndhF-rpl32, trnL_UAG-rpl32, ndhE-ndhG, ndhE-psaC, matK-rps16, ycf1-trnN_GUU, rps2-atpI, cemA-petA* and *petN-psbM* were candidate markers for *Bidens* species' separation. In addition, we tested them by in silico PCR and found *nhD-ccsA*, *ndhI-ndhG*, *ndhF-rpl32*, *trnL_UAG-rpl32*, *ndhE-psaC*, *matK-rps16*, *rps2-atpI*, *cemA-petA*, and *petN-psbM* were highly confident candidates. However, further study based on wet-experiment should be conducted to get the exact conclusion.

It seems that *ndhE, ndhF, ndhG,* and *rpl32* in *Bidens* were under positive selection while the majority of genes were under purifying selection, which is logically sound as CP genes are functional important for photosynthesis. Additionally, we found *Bidens* in China clustered together but formed monophyletic clade with other *Bidens* species from phylogenetic analysis, indicating *Bidens* species might are highly adaptive species to the changing environment world-widely.

## Conclusions

CP genomes are helpful in revealing intra-species relationships, but also in identification of closely-related inter-species identification. In this study, we assembled CP genomes for 3 *Bidens* species endemic to China and conducted CP-genome-widely comparison thoroughly. The genome structure variation, IR expansion and contraction, phylogenetic relationships, mutation hotspot and candidate molecular markers found in this study would provide useful information for genetic diversity analysis and molecular identification for *Bidens* species.

## Supplementary Information

**Additional file 1:** **Table S1.** Length of exons and introns for genes in *Bidens* CP genomes. **Table S2.** Relative Synonymous Codon Usage (RSCU) of *Bidens* CP genomes. **Table S3A.** Number of different SSR types detected in three *Bidens* species. **Table S3B.** Frequency of identified SSR motifs in different repeat class types. **Table S4.** The nucleotide diversity of Genes. **Table S5.** Design primers for *Bidens* species. **Table S6.** *In silico* PCR analysis of the *Bidens *markers. **Table S7.** *Bidens *species from NCBI. **Table S8.** Sequencing raw data information of *Bidens*. **Fig. S1.** ITS1 phylogenetic tree constructed by the maximum parsimony of Bidens. **Fig. S2.** ITS2 phylogenetic tree constructed by the maximum parsimony of Bidens. **Fig. S3.** Gene map of *B. bipinnata* chloroplast genome. **Fig. S4.** Gene map of *B. alba* var. *radiata* chloroplast genome. **Fig. S5A.** Sliding window analysis based on the *Bidens* CP genomes. **Fig. S5B**. The nucleotide diversity (Pi) values of the cp genomes: Pi values of coding genes and Pi values of IGS.

## Data Availability

The authors affirm that all data sets used and analyzed during this study are included in this published article. All the data have been deposited into the GenBank of the National Center of Biotechnology Information under accession number MZ127828 (*Bidens pilosa*), MZ127827 (*Bidens bipinnata*) and MZ127826 *(Bidens alba*).

## Abbreviations

CP: Chloroplast
LSC: Large single-copy region
SSC: Small single-copy region
IR: Inverted repeat region
CDS: Coding DNA sequence
tRNAs: Transport RNAs
rRNAs: Ribosomal RNAs
AT1: AT content in first codon positions of protein coding genes
AT2: AT content in second codon positions of protein coding genes
AT3: AT content in third codon positions of protein coding genes
RSCU: Relative synonymous codon usage
SSRs: Simple sequence repeats
Pi: Nucleotide diversity
Ka/Ks: The rate of nonsynonymous substitutions to the rate of synonymous substitutions

## References

[1] Knope ML, Funk VA, Johnson MA (2020). Dispersal and adaptive radiation of Bidens (Compositae) across the remote archipelagoes of Polynesia. J Syst Evol.

[2] Sun Y, Zhou Q, Wang L (2009). Cadmium tolerance and accumulation characteristics of Bidens pilosa L. as a potential Cd-hyperaccumulator. J Hazard Mater.

[3] Li X, Tian L, Li B (2022). Polyaspartic acid enhances the Cd phytoextraction efficiency of Bidens pilosa by remolding the rhizospheric environment and reprogramming plant metabolism. Chemosphere.

[4] Xuan TD, Khanh TD (2016). Chemistry and pharmacology of Bidens pilosa: an overview. J Pharm Investig.

[5] Bartolome AP, Villasenor IM, Yang WC (2013). Bidens pilosa L. (Asteraceae): Botanical Properties, Traditional Uses, Phytochemistry, and Pharmacology. Evid Based Complement Alternat Med.

[6] Wang R, Wu QX, Shi YP (2010). Polyacetylenes and flavonoids from the aerial parts of Bidens pilosa. Planta Med.

[7] Cai FJ, Li CH, Sun XH (2022). A new dihydroflavone and a new polyacetylene glucoside from Bidens parviflora. J Asian Nat Prod Res.

[8] Yan Z, Chen Z, Zhang L (2022). Bioactive polyacetylenes from Bidens pilosa L and their anti-inflammatory activity. Nat Prod Res.

[9] Bellinger MR, Datlof EM, Selph KE (2022). A Genome for Bidens hawaiensis: A Member of a Hexaploid Hawaiian Plant Adaptive Radiation. J Hered.

[10] Knope ML, Bellinger MR, Datlof EM, Gallaher TJ, Johnson MA (2020). Insights into the Evolutionary History of the Hawaiian Bidens (Asteraceae) Adaptive Radiation Revealed Through Phylogenomics. J Hered.

[11] Wu ZY, Raven PH (2011). Flora of China Science Press (Beijing).

[12] Tsai LC, Wang JC, Hsieh HM (2008). Bidens identification using the noncoding regions of chloroplast genome and nuclear ribosomal DNA. Forensic Sci Int Genet.

[13] Wu LW, Nie LP, Guo SY (2022). Identification of Medicinal Bidens plants for quality control based on organelle gsenomes. Front Pharmacol.

[14] Palmer JD (1985). Comparative organization of chloroplast genomes. Annu Rev Genet.

[15] Logan DC (2006). The mitochondrial compartment. J Exp Bot.

[16] Gong L, Ding X, Guan W (2022). Comparative chloroplast genome analyses of Amomum: insights into evolutionary history and species identification. BMC Plant Biol.

[17] Song Y, Zhao W, Xu J (2022). Chloroplast Genome Evolution and Species Identification of Styrax (Styracaceae). Biomed Res Int.

[18] Cao DL, Zhang XJ, Xie SQ (2022). Application of chloroplast genome in the identification of Traditional Chinese Medicine Viola philippica. BMC Genomics.

[19] Zhou J, Chen X, Cui Y (2017). Molecular Structure and Phylogenetic Analyses of Complete Chloroplast Genomes of Two Aristolochia Medicinal Species. Int J Mol Sci.

[20] Wingett SW, Andrews S (2018). FastQ Screen: A tool for multi-genome mapping and quality control. F1000Res.

[21] Bolger AM, Lohse M, Usadel B (2014). Trimmomatic: a flexible trimmer for Illumina sequence data. Bioinformatics.

[22] Luo RB, Liu BH, Xie YL (2012). SOAPdenovo2: an empirically improved memory-efficient short-read de novo assembler. Gigascience.

[23] Boetzer M, Henkel CV, Jansen HJ (2011). Scaffolding pre-assembled contigs using SSPACE. Bioinformatics.

[24] Nadalin F, Vezzi F, Policriti A (2012). GapFiller: a de novo assembly approach to fill the gap within paired reads. BMC Bioinformatics.

[25] Shi L, Chen H, Jiang M (2019). CPGAVAS2, an integrated plastome sequence annotator and analyzer. Nucleic Acids Res.

[26] Lewis SE, Searle SMJ, Harris N, et al. Apollo: a sequence annotation editor. Genome Biol. 2002;3(12):research0082.1-0082.14. 10.1186/gb-2002-3-12-research0082 PMC151184 12537571

[27] Lowe TM, Chan PP (2016). tRNAscan-SE On-line: integrating search and context for analysis of transfer RNA genes. Nucleic Acids Res.

[28] Greiner S, Lehwark P, Bock R. OrganellarGenomeDRAW (OGDRAW) version 1.3.1: expanded toolkit for the graphical visualization of organellar genomes. Nucleic Acids Res. 2019;47(W1):W59–64. 10.1093/nar/gkz238 PMC6602502 30949694

[29] Mazumdar P, Binti Othman R, Mebus K (2017). Codon usage and codon pair patterns in non-grass monocot genomes. Ann Bot.

[30] Beier S, Thiel T, Munch T (2017). MISA-web: a web server for microsatellite prediction. Bioinformatics.

[31] Kurtz S, Choudhuri JV, Ohlebusch E (2001). REPuter: the manifold applications of repeat analysis on a genomic scale. Nucleic Acids Res.

[32] Charif D, Thioulouse J, Lobry JR (2005). Online synonymous codon usage analyses with the ade4 and seqinR packages. Bioinformatics.

[33] Amiryousefi A, Hyvönen J, Poczai P (2018). IRscope: an online program to visualize the junction sites of chloroplast genomes. Bioinformatics.

[34] Katoh K, Rozewicki J, Yamada KD (2019). MAFFT online service: multiple sequence alignment, interactive sequence choice and visualization. Brief Bioinform.

[35] Rozas J, Ferrer-Mata A, Sanchez-DelBarrio JC (2017). DnaSP 6: DNA Sequence polymorphism analysis of large data sets. Mol Biol Evol.

[36] Frazer KA, Pachter L, Poliakov A (2004). VISTA: computational tools for comparative genomics. Nucleic Acids Res.

[37] Kozlov AM, Darriba D, Flouri T (2019). RAxML-NG: a fast, scalable and user-friendly tool for maximum likelihood phylogenetic inference. Bioinformatics.

[38] Norgate M, Chamings J, Pavlova A (2009). Mitochondrial DNA indicates late pleistocene divergence of populations of Heteronympha merope, an emerging model in environmental change biology. PLoS ONE.

[39] Zhang Z, Xiao J, Wu J (2012). ParaAT: a parallel tool for constructing multiple protein-coding DNA alignments. Biochem Biophys Res Commun.

[40] Zhang Z, Li J, Zhao XQ (2006). KaKs_Calculator: Calculating Ka and Ks Through Model Selection and Model Averaging. Geno, Pro Bioinfo.

[41] Parvathy ST, Udayasuriyan V, Bhadana V (2022). Codon usage bias. Mol Biol Rep.

[42] Jiang H, Tian J, Yang J (2022). Comparative and phylogenetic analyses of six Kenya Polystachya (Orchidaceae) species based on the complete chloroplast genome sequences. BMC Plant Biol.

[43] Park H, Sa KJ, Hyun DY (2021). Identifying SSR Markers Related to Seed Fatty Acid Content in Perilla Crop (Perilla frutescens L.). Plants.

[44] Bhattarai G, Shi A, Kandel DR (2021). Genome-wide simple sequence repeats (SSR) markers discovered from whole-genome sequence comparisons of multiple spinach accessions. Sci Rep.

[45] Nashima K, Hosaka F, Terakami S (2020). SSR markers developed using next-generation sequencing technology in pineapple, Ananas comosus (L.) Merr. Breed Sci.

[46] Li X, Qiao L, Chen B (2022). SSR markers development and their application in genetic diversity evaluation of garlic (Allium sativum) germplasm. Plant Divers.

[47] Kalendar R, Muterko A, Shamekova M (2017). In silico PCR tools for a fast primer, probe, and advanced searching. Methods Mol Biol.

[48] Birky CW (1995). Uniparental inheritance of mitochondrial and chloroplast genes: mechanisms and evolution. Proc Natl Acad Sci U S A.

[49] Lan Z, Shi Y, Yin Q, et al. Comparative and phylogenetic analysis of complete chloroplast genomes from five Artemisia species. Front Plant Sci. 2022;13:1049209:1-9. 10.3389/fpls.2022.1049209 PMC9720176 36479523

[50] Peng JY, Zhang XS, Zhang DG (2022). Newly reported chloroplast genome of Sinosenecio albonervius Y. Liu & Q. E. Yang and comparative analyses with other Sinosenecio species. BMC Genomics.

[51] Yan K, Ran J, Bao S (2022). The complete chloroplast genome sequence of Eupatorium fortunei: genome organization and comparison with related species. Genes (Basel).

[52] Wang YH, Wicke S, Wang H (2018). Plastid Genome Evolution in the Early-Diverging Legume Subfamily Cercidoideae (Fabaceae). Front Plant Sci.

[53] Li C, Zhao Y, Xu Z (2020). Initial Characterization of the Chloroplast Genome of Vicia sepium, an Important Wild Resource Plant, and Related Inferences About Its Evolution. Front Genet.

[54] Xiong Y, Xiong Y, Shu X (2022). Molecular Phylogeography and Intraspecific Divergences in Siberian Wildrye (Elymus sibiricus L.) Wild Populations in China, Inferred From Chloroplast DNA Sequence and cpSSR Markers. Front Plant Sci.

[55] Singh RB, Mahenderakar MD, Jugran AK (2020). Assessing genetic diversity and population structure of sugarcane cultivars, progenitor species and genera using microsatellite (SSR) markers. Gene.

[56] Dodsworth S, Leitch AR, Leitch IJ (2015). Genome size diversity in angiosperms and its influence on gene space. Curr Opin Genet Dev.

