# Supplementary material for: Analysis of the chloroplast genome and phylogenetic evolution of Bidens pilosa
Source: BMC Genomics. 2023 Mar 14;24:113. doi: 10.1186/s12864-023-09195-7 (PMC10015693; doi:10.1186/s12864-023-09195-7)
Supplement: Supplementary file 1 — Additional file 1: Table S1. Length of exons and introns for genes in Bidens CP genomes. Table S2. Relative Synonymous Codon Usage (RSCU) of Bidens CP genomes. Table S3A. Number of different SSR types detected in three Bidens species. Table S3B. Frequency of identified SSR motifs in different repeat class types. Table S4. The nucleotide diversity of Genes. Table S5. Design primers for Bidens species. Table S6. In silico PCR analysis of the Bidens markers. Table S7. Bidens species from NCBI. Table S8. Sequencing raw data information of Bidens. Fig. S1. ITS1 phylogenetic tree constructed by the maximum parsimony of Bidens. Fig. S2. ITS2 phylogenetic tree constructed by the maximum parsimony of Bidens. Fig. S3. Gene map of B. bipinnata chloroplast genome. Fig. S4. Gene map of B. alba var. radiata chloroplast genome. Fig. S5A. Sliding window analysis based on the Bidens CP genomes. Fig. S5B. The nucleotide diversity (Pi) values of the cp genomes: Pi values of coding genes and Pi values of IGS. [file 12864_2023_9195_MOESM1_ESM.docx]

**Table S1.** Length of exons and introns for genes in *Bidens* CP genomes

| Gene | location | ExonI | IntronI | ExonII | IntronII | ExonIII |
| --- | --- | --- | --- | --- | --- | --- |
| trnA-UGC | IR | 37 | 830 | 36 |  |  |
| trnC-GCA | LSC | 38 | 568 | 56 |  |  |
| trnE-UUC | IR | 33 | 779 | 40 |  |  |
| trnK-UUU | LSC | 38 | 2517 | 36 |  |  |
| trnL-UAA | LSC | 35 | 438 | 50 |  |  |
| trnS-CGA | LSC | 31 | 711 | 60 |  |  |
| atpF | LSC | 145 | 701 | 410 |  |  |
| ndhB | IR | 777 | 670 | 756 |  |  |
| petB | LSC | 2 | 921 | 643 |  |  |
| rpl2 | IR | 391 | 653 | 446 | 60 | 291 |
| rps12^*^ | LSC | 391 | 653 | 155 |  |  |
| rpoC1 | LSC | 432 | 736 | 1638 |  |  |
| rps16 | LSC | 40 | 885 | 227 |  |  |
| ycf2 | IR | 5889 | 66 | 543 |  |  |
| ycf3 | LSC | 124 | 699 | 230 | 755 | 153 |
| clpP^#^ | LSC | 71 | 788 | 294 | 637 | 226 |
| psbD^&^ | LSC | 396 | 84 | 666 |  |  |

^*^different in *B. bipinnata*, ^#^ absent from *B. bipinnata*, ^&^only exist in *B. pilosa*

* The rps12 gene is a trans-spliced gene with the 5’ end located in the LSC region and the duplicated 3’ ends in the IR regions.

**Table S2.** Relative Synonymous Codon Usage (RSCU) of *Bidens* CP genomes

| Species | Amino acid | codon | No. | Frequency | RSCU | Amino acid | codon | No. | Frequency | RSCU |
| --- | --- | --- | --- | --- | --- | --- | --- | --- | --- | --- |
| *B. pilosa* | Lys | aaa | 1026 | 0.04 | 1.47 | Glu | gaa | 969 | 0.04 | 1.49 |
|  | Asn | aac | 261 | 0.01 | 0.43 | Asp | gac | 217 | 0.01 | 0.42 |
|  | Lys | aag | 370 | 0.01 | 0.53 | Glu | gag | 328 | 0.01 | 0.51 |
|  | Asn | aat | 958 | 0.04 | 1.57 | Asp | gat | 820 | 0.03 | 1.58 |
|  | Thr | aca | 394 | 0.02 | 1.24 | Ala | gca | 396 | 0.02 | 1.14 |
|  | Thr | acc | 230 | 0.01 | 0.72 | Ala | gcc | 229 | 0.01 | 0.66 |
|  | Thr | acg | 132 | 0.01 | 0.42 | Ala | gcg | 159 | 0.01 | 0.46 |
|  | Thr | act | 514 | 0.02 | 1.62 | Ala | gct | 604 | 0.02 | 1.74 |
|  | Arg | aga | 475 | 0.02 | 1.87 | Gly | gga | 681 | 0.03 | 1.59 |
|  | Ser | agc | 116 | 0.00 | 0.36 | Gly | ggc | 200 | 0.01 | 0.47 |
|  | Arg | agg | 157 | 0.01 | 0.62 | Gly | ggg | 298 | 0.01 | 0.70 |
|  | Ser | agt | 394 | 0.02 | 1.21 | Gly | ggt | 533 | 0.02 | 1.25 |
|  | Ile | ata | 690 | 0.03 | 0.97 | Val | gta | 499 | 0.02 | 1.49 |
|  | Ile | atc | 422 | 0.02 | 0.59 | Val | gtc | 178 | 0.01 | 0.53 |
|  | Met | atg | 608 | 0.02 | 1.00 | Val | gtg | 186 | 0.01 | 0.55 |
|  | Ile | att | 1030 | 0.04 | 1.44 | Val | gtt | 479 | 0.02 | 1.43 |
|  | Gln | caa | 684 | 0.03 | 1.52 | Stp | taa | 47 | 0.00 | 1.66 |
|  | His | cac | 136 | 0.01 | 0.46 | Tyr | tac | 176 | 0.01 | 0.37 |
|  | Gln | cag | 217 | 0.01 | 0.48 | Stp | tag | 23 | 0.00 | 0.81 |
|  | His | cat | 454 | 0.02 | 1.54 | Tyr | tat | 776 | 0.03 | 1.63 |
|  | Pro | cca | 311 | 0.01 | 1.16 | Ser | tca | 414 | 0.02 | 1.27 |
|  | Pro | ccc | 189 | 0.01 | 0.70 | Ser | tcc | 304 | 0.01 | 0.93 |
|  | Pro | ccg | 162 | 0.01 | 0.60 | Ser | tcg | 150 | 0.01 | 0.46 |
|  | Pro | cct | 411 | 0.02 | 1.53 | Ser | tct | 582 | 0.02 | 1.78 |
|  | Arg | cga | 342 | 0.01 | 1.34 | Stp | tga | 15 | 0.00 | 0.53 |
|  | Arg | cgc | 98 | 0.00 | 0.39 | Cys | tgc | 80 | 0.00 | 0.56 |
|  | Arg | cgg | 116 | 0.00 | 0.46 | Trp | tgg | 427 | 0.02 | 1.00 |
|  | Arg | cgt | 339 | 0.01 | 1.33 | Cys | tgt | 206 | 0.01 | 1.44 |
|  | Leu | cta | 361 | 0.01 | 0.80 | Leu | tta | 847 | 0.03 | 1.88 |
|  | Leu | ctc | 188 | 0.01 | 0.42 | Phe | ttc | 481 | 0.02 | 0.67 |
|  | Leu | ctg | 171 | 0.01 | 0.38 | Leu | ttg | 557 | 0.02 | 1.24 |
|  | Leu | ctt | 580 | 0.02 | 1.29 | Phe | ttt | 951 | 0.04 | 1.33 |
| *B. bipinnata* | Lys | aaa | 1025 | 0.04 | 1.46 | Glu | gaa | 982 | 0.04 | 1.51 |
|  | Asn | aac | 277 | 0.01 | 0.45 | Asp | gac | 221 | 0.01 | 0.42 |
|  | Lys | aag | 377 | 0.01 | 0.54 | Glu | gag | 320 | 0.01 | 0.49 |
|  | Asn | aat | 962 | 0.04 | 1.55 | Asp | gat | 820 | 0.03 | 1.58 |
|  | Thr | aca | 386 | 0.02 | 1.21 | Ala | gca | 393 | 0.02 | 1.12 |
|  | Thr | acc | 235 | 0.01 | 0.74 | Ala | gcc | 231 | 0.01 | 0.66 |
|  | Thr | acg | 129 | 0.01 | 0.40 | Ala | gcg | 158 | 0.01 | 0.45 |
|  | Thr | act | 525 | 0.02 | 1.65 | Ala | gct | 625 | 0.02 | 1.78 |
|  | Arg | aga | 470 | 0.02 | 1.84 | Gly | gga | 684 | 0.03 | 1.58 |
|  | Ser | agc | 119 | 0.00 | 0.36 | Gly | ggc | 201 | 0.01 | 0.46 |
|  | Arg | agg | 154 | 0.01 | 0.60 | Gly | ggg | 295 | 0.01 | 0.68 |
|  | Ser | agt | 403 | 0.02 | 1.21 | Gly | ggt | 551 | 0.02 | 1.27 |
|  | Ile | ata | 677 | 0.03 | 0.95 | Val | gta | 508 | 0.02 | 1.49 |
|  | Ile | atc | 433 | 0.02 | 0.60 | Val | gtc | 180 | 0.01 | 0.53 |
|  | Met | atg | 611 | 0.02 | 1.00 | Val | gtg | 187 | 0.01 | 0.55 |
|  | Ile | att | 1039 | 0.04 | 1.45 | Val | gtt | 488 | 0.02 | 1.43 |
|  | Gln | caa | 683 | 0.03 | 1.51 | Stp | taa | 48 | 0.00 | 1.69 |
|  | His | cac | 144 | 0.01 | 0.48 | Tyr | tac | 177 | 0.01 | 0.37 |
|  | Gln | cag | 220 | 0.01 | 0.49 | Stp | tag | 23 | 0.00 | 0.81 |
|  | His | cat | 453 | 0.02 | 1.52 | Tyr | tat | 781 | 0.03 | 1.63 |
|  | Pro | cca | 311 | 0.01 | 1.15 | Ser | tca | 409 | 0.02 | 1.23 |
|  | Pro | ccc | 192 | 0.01 | 0.71 | Ser | tcc | 306 | 0.01 | 0.92 |
|  | Pro | ccg | 159 | 0.01 | 0.59 | Ser | tcg | 153 | 0.01 | 0.46 |
|  | Pro | cct | 416 | 0.02 | 1.54 | Ser | tct | 603 | 0.02 | 1.82 |
|  | Arg | cga | 344 | 0.01 | 1.34 | Stp | tga | 14 | 0.00 | 0.49 |
|  | Arg | cgc | 97 | 0.00 | 0.38 | Cys | tgc | 75 | 0.00 | 0.53 |
|  | Arg | cgg | 119 | 0.00 | 0.46 | Trp | tgg | 434 | 0.02 | 1.00 |
|  | Arg | cgt | 352 | 0.01 | 1.38 | Cys | tgt | 208 | 0.01 | 1.47 |
|  | Leu | cta | 373 | 0.01 | 0.82 | Leu | tta | 854 | 0.03 | 1.88 |
|  | Leu | ctc | 189 | 0.01 | 0.42 | Phe | ttc | 499 | 0.02 | 0.69 |
|  | Leu | ctg | 164 | 0.01 | 0.36 | Leu | ttg | 561 | 0.02 | 1.24 |
|  | Leu | ctt | 581 | 0.02 | 1.28 | Phe | ttt | 950 | 0.04 | 1.31 |
| *B. alba* | Lys | aaa | 1027 | 0.04 | 1.47 | Glu | gaa | 986 | 0.04 | 1.50 |
|  | Asn | aac | 271 | 0.01 | 0.44 | Asp | gac | 220 | 0.01 | 0.42 |
|  | Lys | aag | 371 | 0.01 | 0.53 | Glu | gag | 329 | 0.01 | 0.50 |
|  | Asn | aat | 968 | 0.04 | 1.56 | Asp | gat | 825 | 0.03 | 1.58 |
|  | Thr | aca | 392 | 0.02 | 1.22 | Ala | gca | 402 | 0.02 | 1.13 |
|  | Thr | acc | 238 | 0.01 | 0.74 | Ala | gcc | 232 | 0.01 | 0.65 |
|  | Thr | acg | 131 | 0.01 | 0.41 | Ala | gcg | 158 | 0.01 | 0.44 |
|  | Thr | act | 524 | 0.02 | 1.63 | Ala | gct | 632 | 0.02 | 1.78 |
|  | Arg | aga | 476 | 0.02 | 1.85 | Gly | gga | 688 | 0.03 | 1.58 |
|  | Ser | agc | 119 | 0.00 | 0.36 | Gly | ggc | 207 | 0.01 | 0.47 |
|  | Arg | agg | 161 | 0.01 | 0.63 | Gly | ggg | 298 | 0.01 | 0.68 |
|  | Ser | agt | 402 | 0.02 | 1.21 | Gly | ggt | 553 | 0.02 | 1.27 |
|  | Ile | ata | 687 | 0.03 | 0.95 | Val | gta | 511 | 0.02 | 1.49 |
|  | Ile | atc | 437 | 0.02 | 0.60 | Val | gtc | 179 | 0.01 | 0.52 |
|  | Met | atg | 620 | 0.02 | 1.00 | Val | gtg | 188 | 0.01 | 0.55 |
|  | Ile | att | 1043 | 0.04 | 1.44 | Val | gtt | 493 | 0.02 | 1.44 |
|  | Gln | caa | 691 | 0.03 | 1.52 | Stp | taa | 48 | 0.00 | 1.67 |
|  | His | cac | 144 | 0.01 | 0.48 | Tyr | tac | 179 | 0.01 | 0.37 |
|  | Gln | cag | 219 | 0.01 | 0.48 | Stp | tag | 23 | 0.00 | 0.80 |
|  | His | cat | 454 | 0.02 | 1.52 | Tyr | tat | 786 | 0.03 | 1.63 |
|  | Pro | cca | 314 | 0.01 | 1.16 | Ser | tca | 413 | 0.02 | 1.25 |
|  | Pro | ccc | 192 | 0.01 | 0.71 | Ser | tcc | 304 | 0.01 | 0.92 |
|  | Pro | ccg | 160 | 0.01 | 0.59 | Ser | tcg | 151 | 0.01 | 0.46 |
|  | Pro | cct | 419 | 0.02 | 1.54 | Ser | tct | 599 | 0.02 | 1.81 |
|  | Arg | cga | 343 | 0.01 | 1.33 | Stp | tga | 15 | 0.00 | 0.52 |
|  | Arg | cgc | 99 | 0.00 | 0.38 | Cys | tgc | 80 | 0.00 | 0.56 |
|  | Arg | cgg | 115 | 0.00 | 0.45 | Trp | tgg | 438 | 0.02 | 1.00 |
|  | Arg | cgt | 350 | 0.01 | 1.36 | Cys | tgt | 207 | 0.01 | 1.44 |
|  | Leu | cta | 372 | 0.01 | 0.82 | Leu | tta | 852 | 0.03 | 1.87 |
|  | Leu | ctc | 189 | 0.01 | 0.41 | Phe | ttc | 501 | 0.02 | 0.69 |
|  | Leu | ctg | 171 | 0.01 | 0.38 | Leu | ttg | 565 | 0.02 | 1.24 |
|  | Leu | ctt | 586 | 0.02 | 1.29 | Phe | ttt | 956 | 0.04 | 1.31 |

**Table S3A. Number of different SSR types detected in three *Bidens* species**

| Type | Number | | |
| --- | --- | --- | --- |
|  | *B. pilosa* | *B. bipinnata* | *B. alba* |
| A | 15 | 17 | 18 |
| C | 2 | 1 | 1 |
| G | 2 | 0 | 0 |
| T | 20 | 24 | 23 |
| AT | 2 | 4 | 4 |
| TA | 3 | 3 | 3 |
| TC | 1 | 1 | 1 |
| AAG | 1 | 1 | 1 |
| CTT | 1 | 1 | 1 |
| TAA | 1 | 1 | 1 |
| TTA | 0 | 1 | 1 |
| TTC | 1 | 1 | 1 |
| AATA | 1 | 1 | 1 |
| AATC | 1 | 1 | 1 |
| ATAG | 1 | 0 | 0 |
| ATTT | 1 | 1 | 1 |
| GATA | 1 | 1 | 1 |
| TATT | 1 | 1 | 1 |
| TTTA | 1 | 2 | 1 |
| TTTC | 2 | 2 | 2 |
| ATCTT | 1 | 1 | 1 |
| ATTTT | 1 | 1 | 1 |
| ATTGAA | 1 | 0 | 0 |

**Table S3B. Frequency of identified SSR motifs in different repeat class types**

| Type | Number | | |
| --- | --- | --- | --- |
|  | *B. pilosa* | *B. bipinnata* | *B. alba* |
| A/T | 35 | 41 | 41 |
| C/G | 4 | 1 | 1 |
| AG/CT | 1 | 1 | 1 |
| AT/AT | 5 | 7 | 7 |
| AAG/CTT | 3 | 3 | 3 |
| AAT/ATT | 1 | 2 | 2 |
| AAAG/CTTT | 2 | 2 | 2 |
| AAAT/ATTT | 4 | 5 | 4 |
| AATC/ATTG | 1 | 1 | 1 |
| AGAT/ATCT | 2 | 1 | 1 |
| AAAAT/ATTTT | 1 | 1 | 1 |
| AAGAT/ATCTT | 1 | 1 | 1 |
| AAATTG/AATTTC | 1 | 0 | 0 |

**Table S4. The nucleotide diversity of Genes**

| Genes | Pi | Genes | Pi | Genes | Pi |
| --- | --- | --- | --- | --- | --- |
| *petN* | 0.00000 | *ndhJ* | 0.00414 | *rps8* | 0.00635 |
| *psbI* | 0.00000 | *psbJ* | 0.00429 | *ndhI* | 0.00646 |
| *rpl36* | 0.00000 | *petL* | 0.00430 | *rbcL* | 0.00650 |
| *psbF* | 0.00000 | *rps4* | 0.00433 | *ndhG* | 0.00660 |
| *psbZ* | 0.00072 | *psbK* | 0.00441 | *psaI* | 0.00670 |
| *rpl14* | 0.00073 | *rpl33* | 0.00443 | *ndhC* | 0.00693 |
| *ycf3* | 0.00106 | *atpA* | 0.00446 | *accD* | 0.00693 |
| *psbE* | 0.00107 | *rpoB* | 0.00462 | *rpoC2* | 0.00695 |
| *atpH* | 0.00165 | *atpB* | 0.00471 | *cemA* | 0.00718 |
| *psaA* | 0.00228 | *atpF* | 0.00497 | *rps16* | 0.00743 |
| *psbC* | 0.00263 | *ndhE* | 0.00509 | *ndhD* | 0.00755 |
| *psaB* | 0.00283 | *petA* | 0.00510 | *psbH* | 0.00757 |
| *ycf4* | 0.00286 | *rpl16* | 0.00514 | *psbM* | 0.00766 |
| *psbN* | 0.00310 | *rps3* | 0.00521 | *rpl22* | 0.00820 |
| *rps11* | 0.00327 | *rps19* | 0.00531 | *rps15* | 0.00856 |
| *rpl32* | 0.00329 | *psaJ* | 0.00542 | *rps18* | 0.00880 |
| *petG* | 0.00343 | *ndhK* | 0.00547 | *psaC* | 0.00901 |
| *rps2* | 0.00348 | *petB* | 0.00552 | *ndhF* | 0.00905 |
| *ndhH* | 0.00352 | *psbT* | 0.00558 | *ccsA* | 0.00909 |
| *atpE* | 0.00396 | *rps14* | 0.00597 | *matK* | 0.00911 |
| *rpoC1* | 0.00401 | *atpI* | 0.00612 | *ndhA* | 0.01011 |
| *infA* | 0.00412 | *petD* | 0.00619 | *rpl20* | 0.02101 |
| *psbB* | 0.00412 |  |  |  |  |

**Table S5.** **Design primers for *Bidens* species**

|  | primer | F-sequence（5'-3'） | R-sequence（5'-3'） |
| --- | --- | --- | --- |
| 1 | *ndhD-ccsA* | TACTGTAATGCGCCTCTCCG | AGTCGTATCCTGTGGCTTTGG |
| 2 | *ndhI-ndhG* | ATCCATTCTCAGCCGCGGTA | GCAGAAGAAGAGTTAGTAGCAGG |
| 3 | *ndhF-rpl32* | GAGTTCGAGCAAGCCGCTAT | CGGACTCGAACCGAGATGC |
| 4 | *trnL_UAG-rpl32* | CCAAGGCTTTGCTACTTCGG | AAGGCCCAAGTTTCCTTTGGA |
| 5 | *ndhE-ndhG* | AACACAATTCTCGCCGCCT | TCAAATTGCTTTGGGTCGGTT |
| 6 | *ndhE-psaC* | CCACGGTCCAAAGACGGAAA | ATTAGGGGGTCTGGGAGTAGT |
| 7 | *matK-rps16* | AGCCGAGTACTCTACCGTTG | TGATGGGTTCGTTGGATTAGC |
| 8 | *ycf1-trnN_GUU* | CAAAGGACTCTGCCCTTCCA | GCGAGACGAGCCGTTTATCA |
| 9 | *rps2-atpI* | TCTGAGAGATCAAAAGGGGCAC | TGACGATATCGAGCTGGAACA |
| 10 | *cemA-petA* | TAGAGTCGACGAATGAGATGGG | AATCTCCGGGATGGGTAGAA |
| 11 | *petN-psbM* | CCGCCGATTATAGCCCGTTG | TACTTTCCGTAAGCGAGCCC |

**Table S6.** *In silico* PCR analysis of the *Bidens* markers

| Alignment option | IGS | Stringency criteria |
| --- | --- | --- |
| Initial searching word size (bp) |  | 11 |
| Important size of 3’-end with mismatches (bp) | | 16 |
| Mismatches allowed in last 15 bases of 3’-end (bp) | | 1 |
| Minimal complement primer Length (bp) |  | 20 |
| Local alignment similarity (%) |  | 85 |
| PCR success (%)^*^ | *ndhD-ccsA* | 100 |
|  | *ndhI-ndhG* | 100 |
|  | *ndhF-rpl32* | 100 |
|  | *trnL_UAG-rpl32* | 100 |
|  | *ndhE-ndhG* | 50 |
|  | *ndhE-psaC* | 100 |
|  | *matk-rps16* | 100 |
|  | *ycf1-trnN_GUU* | 83 |
|  | *rps2-atpI* | 100 |
|  | *cemA-petA* | 100 |
|  | *petN-psbM* | 100 |

^*^Simulated by the *in-silico* PCR procedure in FastPCR program (v6.7) with two criteria.

**Table S7. *Bidens* species from NCBI**

| species | Accession number | Abbreviations | remarks |
| --- | --- | --- | --- |
| *Bidens asymmetric* | NC_047268.1 | Bas |  |
| *Bidens campylotheca* | MN433107.1 | Bca |  |
| *Bidens cervicata* | MN433100.1 | Bce |  |
| *Bidens conjuncta* | NC_047263.1 | Bco |  |
| *Bidens menziesii* | NC_047260.1 | Bme |  |
| *Bidens pachyloma* | NC_047272.1 | Bpa |  |
| *Bidens torta* | NC_047275.1 | Bto |  |
| *Bidens wiebkei* | NC_047262.1 | Bwi |  |
| *Bidens hawaiensis* | NC_047259.1 | Bha |  |
| *Bidens molokaiensis* | MN433097.1 | Bmo |  |
| *Bidens valida* | MN433092.1 | Bva |  |
| *Bidens bipinnata* | MZ127827 | Bbi |  |
| *Bidens pilosa* | MZ127828 | Bpi |  |
| *Bidens alba* | MZ127826 | Bal |  |
| *Helianthus annuus* | CM007907.1 | Han | outgroup |

**Table S8. Sequencing raw data information of *Bidens***

| species | read_num | base_num(Gb) | GC_percent(%) | max_length | min_length |
| --- | --- | --- | --- | --- | --- |
| *Bidens alba* | 43309372 | 6.58 | 36.64 | 151 | 151 |
| *Bidens pilosa* | 53680388 | 8.16 | 36.52 | 151 | 151 |
| *Bidens bipinnata* | 46427538 | 7.06 | 36.98 | 151 | 151 |


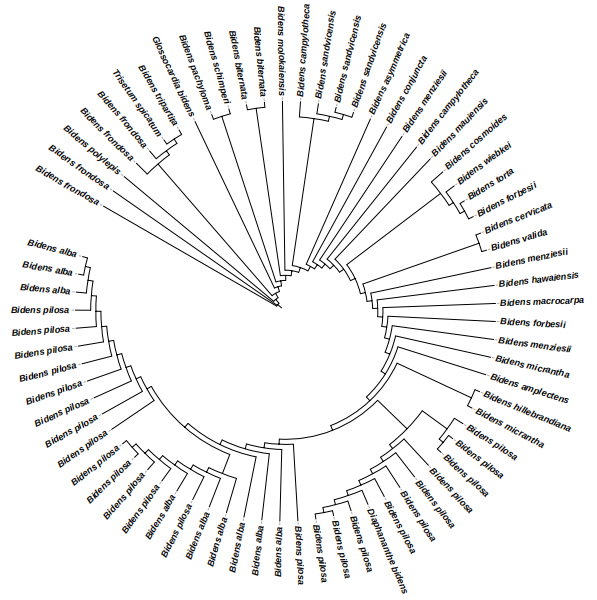


Fig. S1. ITS1 phylogenetic tree constructed by the maximum parsimony of *Bidens*


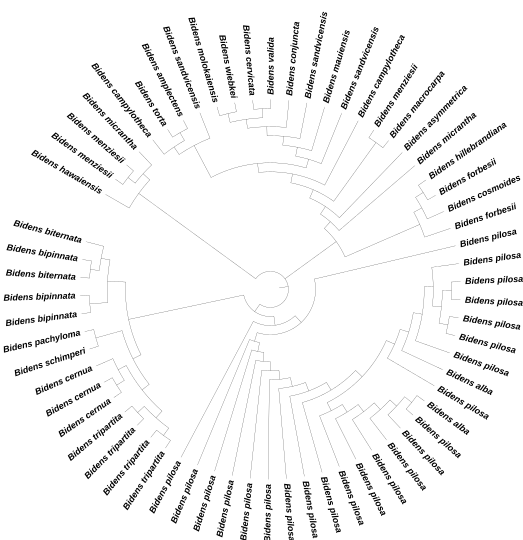


Fig. S2. ITS2 phylogenetic tree constructed by the maximum parsimony of *Bidens*


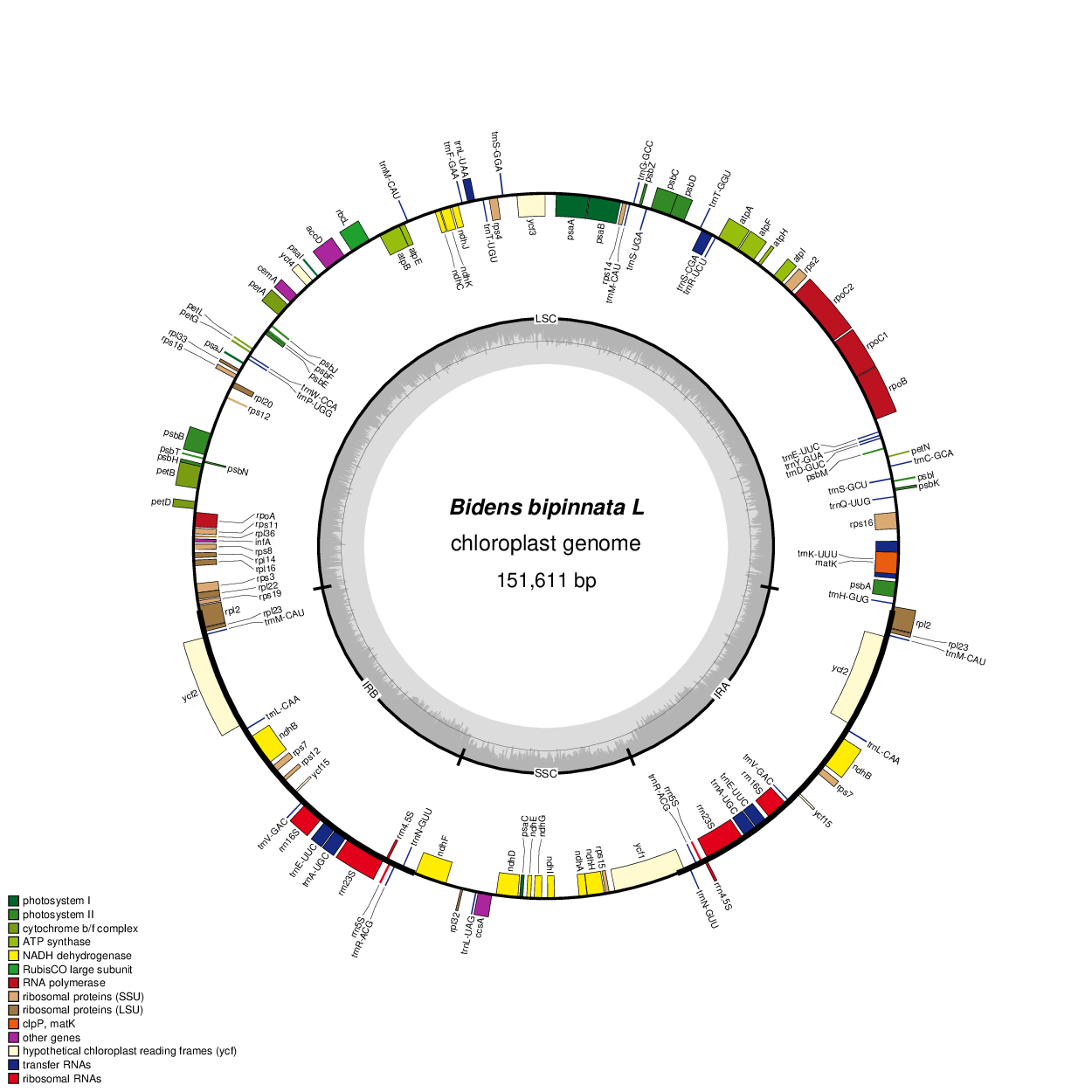


Fig. S3. Gene map of *B. bipinnata* chloroplast genome

Genes drawn within the circle are transcribed clockwise; genes drawn outside are transcribed counterclockwise. Genes in different functional groups are shown in different colors. Dark bold lines indicate the extent of the inverted repeats (IRa and IRb) that separate the genomes into small single-copy (SSC) and large single-copy (LSC) regions.


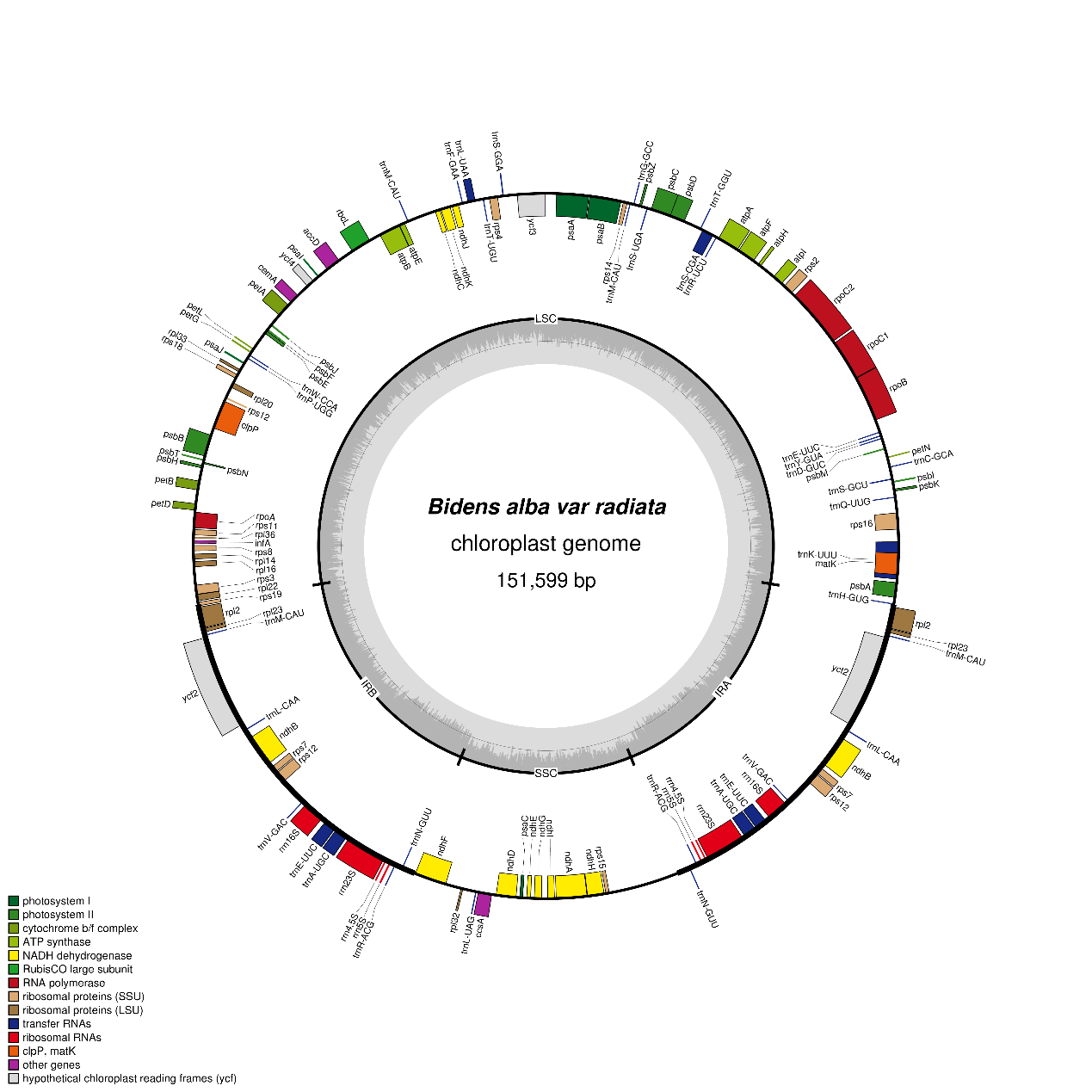


Fig. S4. Gene map of *B. alba* var. *radiata* chloroplast genome

Genes drawn within the circleare transcribed clockwise; genes drawn outside are transcribed counterclockwise. Genesin different functional groups are shown in different colors. Dark bold lines indicate the extent of the inverted repeats (IRa and IRb) that separate the genomes into small single-copy (SSC) and large single-copy (LSC) regions.

| A |
| --- |
| B |
|  |

Fig. S5A. Sliding window analysis based on the *Bidens* CP genomes.

Window length: 800bp; step size: 200 bp. X-axis: position of the midpoint of a window. Y-axis: nucleotide diversity of each window.

Fig. S5B.The nucleotide diversity (Pi) values of the cp genomes: Pi values of coding genes and Pi values of IGS.
